# Spin Bias Is Common in the Abstracts and Main Body of Systematic Reviews and Meta-analyses of Hip Arthroscopy in the Setting of Borderline Hip Dysplasia

**DOI:** 10.1016/j.asmr.2024.100971

**Published:** 2024-07-02

**Authors:** Jeffrey J. Theismann, Matthew J. Hartwell, Samuel G. Moulton, Stephanie E. Wong, Alan L. Zhang

**Affiliations:** Department of Orthopedic Surgery, University of California San Francisco, 1500 Owens Street, San Francisco, California, U.S.A.

## Abstract

**Purpose:**

To assess the quality and presence of spin bias in the abstracts of systematic reviews and meta-analyses that evaluated the outcomes of using hip arthroscopy for the treatment of hip pathology in the setting of borderline hip dysplasia.

**Methods:**

PubMed and Embase were searched using the terms “borderline hip dysplasia” and “systematic review” or “meta-analysis.” Forty-one initial studies were identified, and 12 met the inclusion criteria. Study characteristics were then collected, and each study was evaluated for the 15 most common types of bias and study quality using A Measurement Tool to Assess Systematic Reviews 2 (AMSTAR 2) rating system. Inclusion criteria included a systematic review with or without meta-analysis, published in a peer-reviewed journal, accessible in English, with outcomes after hip arthroscopy for borderline hip dysplasia.

**Results:**

The 12 reviewed studies were published between 2016 and 2023, and 10 of the studies represented Level IV evidence (2 studies were Level III evidence). At least 1 form of spin was identified in 83% (10/12) of the included studies. Regarding the specific categories of spin type, misleading interpretation was identified in 58% (7/12) of the studies, misleading reporting in 67% (8/12) of the studies, and inappropriate extrapolation in 50% (6/12) of the studies. On the basis of the AMSTAR 2 assessment, 92% (11/12) were categorized as either low quality or critically low quality, with 1 study being categorized as moderate.

**Conclusions:**

Spin bias is frequently encountered in the abstracts for systematic reviews and meta-analyses that evaluate outcomes after hip arthroscopy for the treatment of hip pathology in the setting of borderline hip dysplasia.

**Level of Evidence:**

Level IV, systematic review of Level III and IV studies.

Hip arthroscopy has become increasingly used in the United States,^1^ and as the number of procedures has risen, the indications have likewise increased to include those with borderline hip dysplasia, defined most commonly as a lateral center edge angle (LCEA) of between 20° and 25° degrees.^2^ Ricciardi et al.^3^ showed that in the setting of hip dysplasia, as defined by an LCEA less then 20° degrees, treatment with hip arthroscopy followed by delayed periacetabular osteotomy (PAO) demonstrated worse outcomes compared with those who had hip dysplasia treated with initial periacetabular osteotomy and open osteochondroplasty.

Although hip arthroscopy alone has been clearly demonstrated to have no long-term benefit for true hip dysplasia, several authors have found a role for hip arthroscopy alone to improve the outcomes for patients with borderline hip dysplasia, so long as particular care is taken with management of the hip capsule.^4^^,^^5^ Inappropriate management of the capsule can result in iatrogenic injuries ranging from microinstability to frank postoperative hip dislocations.^6^, ^7^, ^8^ There have been an increasing number of systematic reviews and meta-analysis investigating the role of hip arthroscopy in the treatment of borderline hip dysplasia, and these studies can have significant impact on physician practice patterns as they pool previously published data to allow us better understanding. Care must be taken when using systematic reviews and meta-analyses in clinical decision making because there is increasing interest in the presence of spin bias of the abstracts of these studies in the orthopedic literature.^9^^,^^10^

Spin bias is defined as bias in the presentation and interpretation of data that may mislead readers. Yavchitz et al.^11^ categorized spin into 3 broad categories: (1) misleading reporting, (2) misleading interpretation, and (3) inappropriate extrapolation. There has been increasing interest in assessing spin bias in orthopaedic studies as Reddy et al.^12^ found 36% of systematic reviews and meta-analyses of all rotator cuff repair treatments continued spin.

The purpose of this current study was to assess the quality and presence of spin bias in the abstracts of systematic reviews and meta-analyses that evaluated the outcomes of using hip arthroscopy for the treatment of hip pathology in the setting of borderline hip dysplasia. Our hypothesis was that there would be spin bias present and that studies not mentioning their funding source and studies not reporting adherence to PRISMA (Preferred Reporting Items for Systematic Reviews and Meta-Analyses) guidelines will have higher rates of bias.

## Methods

An Embase and PubMed search was performed using the terms “borderline hip dysplasia” and “systematic review” or “meta-analysis.” This study was exempt from institutional review board approval. The inclusion criteria for this study met all the following characteristics: a systematic review involving only human subjects with or without meta-analysis, published in a peer-reviewed journal, accessible in English, with outcomes following hip arthroscopy for borderline hip dysplasia. Articles were excluded for the following reasons: 21 were not related to treatment of borderline dysplasia, 7 were duplicate studies, and 1 did not have the correct study design.

Abstracts were reviewed by 3 authors (M.J.H., J.J.T., A.Z.) to verify inclusion and evaluate spin. From each study, the following data points were obtained: publication year, journal, funding sources, level of evidence, adherence to PRISMA guidelines, primary and secondary outcomes, and 5-year impact factor. The entire article text was evaluated and then compared with results and conclusions presented in the abstract. Each of the study abstracts were then evaluated for spin using the 15 common forms of spin (Table 1), with these forms of spin being grouped into misleading interpretation, misleading reports, and inappropriate extrapolation.^11^

**Table 1:** Frequency of Spin Category and Type in Reviewed Studies

Category and Type	Description	Abstracts With spin, n
Misleading interpretation		7
1	Conclusion contains recommendations for clinical practice not supported by the findings	4
2	Title claims or suggests a beneficial effect of the experimental intervention not supported by the findings	1
4	Conclusion claims safety based on non–statistically significant results with a wide confidence interval	3
9	Conclusion claims the beneficial effect of the experimental treatment despite reporting bias	7
12	Conclusion claims equivalence or comparable effectiveness for non–statistically significant results with a wide confidence interval	6
Misleading reporting		8
3	Selective reporting of or overemphasis on efficacy outcomes or analysis favoring the beneficial effect of the experimental intervention	4
5	Conclusion claims the beneficial effect of the experimental treatment despite high risk of bias in primary studies	7
6	Selective reporting of or overemphasis on harm outcomes or analysis favoring the safety of the experimental intervention	0
10	Authors hide or do not present any conflict of interest	1
11	Conclusion focuses selectively on statistically significant efficacy outcome	0
13	Failure to specify the direction of the effect when it favors the control intervention	0
14	Failure to report a wide confidence interval of estimates	6
Inappropriate extrapolation		6
7	Conclusion extrapolates the review’s findings to a different intervention (i.e., claiming efficacy of one specific intervention although the review covers a class of several interventions)	1
8	Conclusion extrapolates the review’s findings from a surrogate marker or a specific outcome to the global improvement of the disease	5
15	Conclusion extrapolates the review’s findings to a different population or setting	0

As spin bias was assessed in the abstracts, the quality of the systematic reviews was also recorded according to A Measurement Tool to Assess Systematic Reviews, version 2 (AMSTAR 2) criteria. AMSTAR 2 is a tool designed to help clinicians evaluate the quality of systematic reviews before applying those systematic reviews to clinical practice.

Using descriptive statistics, the frequency of spin and each subtype of spin was recorded. An association between each type of spin or the presence of any type of spin and predictors for spin (PRISMA adherence, funding source, and AMSTAR 2 grading) was assessed using the Fisher’s exact test. A *P* value <.05 was considered statistically significant for all calculations. All statistical analyses were performed in Excel for Mac (Version 16.80; Microsoft, Redmond, WA) and JMP Pro (Version 17.0; SAS, Cary, NC).

## Results

**Fig 1:**
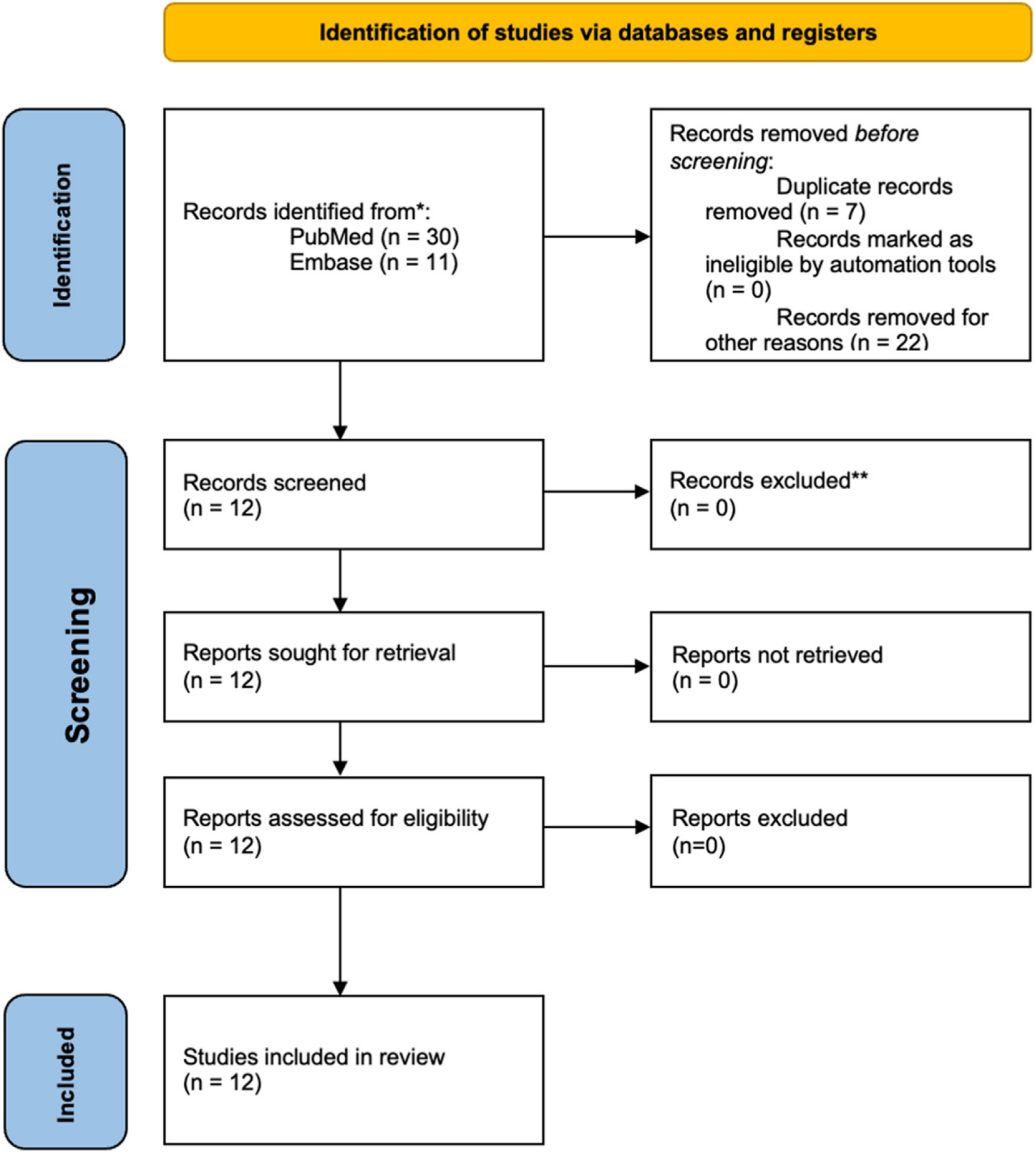
The Preferred Reporting Items for Systematic Review and Meta-Analysis (PRISMA) flow diagram.

The final query was completed in January 2024. This resulted in a total of 41 studies. Of these 41 studies, 30 were identified during a PubMed search and an additional 11 were identified through an Embase (Ovid) search. Of these studies, 21 were excluded because they were not related to borderline hip dysplasia, 7 were duplicates, and 1 was not the appropriate study design for inclusion in our study. A full text screening was performed on the remaining 12 articles, and no further articles were excluded (Fig 1).^13^, ^14^, ^15^, ^16^, ^17^, ^18^, ^19^, ^20^, ^21^, ^22^, ^23^, ^24^ The studies were published between 2016 and 2023. There were 60 studies published between 2003 and 2022 included in the systematic reviews/meta-analyses reviewed. The 12 systematic review/meta-analysis studies were either Level III or Level IV evidence (Table 2). The studies were published in 8 peer-reviewed journals. Level of evidence was reported in all studies, 2 were Level III and included a meta-analysis/systematic review, and the remaining 10 studies were Level IV and were systematic reviews.

**Table 2:** Systematic Reviews and Meta-analyses included

Reference	First Author	Journal	Year	Impact Factor	LOE	Study Design	Intervention	Funding Source	AMSTAR 2 rating	Journal Recomm. to Follow PRISMA?	Reported Adherence to PRISMA Guidelines	Spin Bias Present, Categories Present
13	Krivicich	*JAAOS*	2023	3.2	III	SR & MA	Surgery—HA	Not mentioned	Moderate	No	Yes	Yes, inappropriate extrapolation
14	Lee	*Arthroscopy*	2023	8.5	IV	SR	Surgery—HA	Not mentioned	Critically low	Yes	Yes	Yes, inappropriate extrapolation
15	Murata	*OJSM*	2021	3.5	III	SR & MA	Surgery—HA	Not mentioned	Critically low	Yes	Yes	Yes, misleading reporting, misleading interpretation
16	Murata	*OJSM*	2021	3.5	IV	SR	Surgery—HA vs. PAO	Not mentioned	Low	Yes	Yes	No
17	Kuroda	*Arthroscopy*	2020	8.5	IV	SR	Surgery—HA	Not mentioned	Critically low	Yes	Yes	Yes, misleading reporting, misleading interpretation, inappropriate extrapolation
18	Shah	*KSSTA*	2020	7.3	IV	SR	Surgery—HA	Not funded	Low	Yes	Yes	Yes, misleading reporting, misleading interpretation
19	Adler	*Arthroscopy*	2019	8.5	IV	SR	Surgery—HA, PAO	Not mentioned	Critically low	Yes	Yes	No
20	Ding	*AJSM*	2019	9.8	IV	SR	Surgery—HA	Not mentioned	Critically low	Yes	Yes	Yes, misleading reporting, misleading interpretation, inappropriate extrapolation
21	Barton	*IOJ*	2019	1.5	IV	SR	Surgery—HA, PAO	Not funded	Critically low	No	No	Yes, misleading reporting, misleading interpretation, inappropriate extrapolation
22	Lodhia	*Arthroscopy*	2016	8.5	IV	SR	Surgery—HA, PAO	Not funded	Critically low	Yes	No	Yes, misleading reporting, misleading interpretation, inappropriate extrapolation
23	Jo	*JHPS*	2016	n/a	IV	SR	Surgery—HA	Not funded	Critically low	Yes	Yes	Yes, misleading reporting, misleading interpretation
24	Yeung	*Bone Joint Res*	2016	6.9	IV	SR	Surgery—HA	Not funded	Low	Yes	No	Yes, misleading reporting

*AJSM*, *The American Journal of Sports Medicine*; AMSTAR 2, A Measurement Tool to Assess Systematic Reviews version 2; *Bone Joint Res*, *Bone & Joint Research;* HA, hip arthroscopy; *IOJ*, *The Iowa Orthopaedic Journal*; *JAAOS*, *Journal of the American Academy of Orthopaedic Surgeons*; *JHPS*, *Journal of Hip Preservation Surgery*; *KSSTA*, *Knee Surgery, Sports Traumatology, Arthroscopy*; LOE, level of evidence; MA, meta-analysis; n/a, not applicable; *OJSM*, *Orthopaedic Journal of Sports Medicine*; PAO, periacetabular osteotomy; PRISMA, Preferred Reporting Items for Systematic Reviews and Meta-Analyses; Recomm., recommendation; SR, systematic review.

Of the 8 peer-reviewed journals for which these studies were published 6 of 8 recommended following PRISMA guidelines, and of the 10 studies, 75% (9/12) reported adherence to PRISMA guidelines. Patient-reported outcome changes were the primary endpoint in 92% of studies (11/12) with 92% (11/12) having either a primary or secondary outcome of revision surgery, or conversion to total hip arthroplasty, Of the 12 studies we reviewed, 83% (10/12) had at least 1 form of spin identified. Within the 3 categories of spin, 67% (8/12) had misleading reporting of the studies, 58% (7/12) had misleading interpretation of the studies included, and 50% (6/12) had inappropriate extrapolation of the studies (Table 1). The most common types of spin were type 9 (“conclusion claims that the beneficial effect of the experiment despite reporting bias”) and type 5 (“Conclusion claims the beneficial effect of the experimental treatment despite high risk of bias in primary studies”), which were found in 58% of studies (7/12).

Subgroup analysis showed that 7 of the 12 studies did not mention their funding source. Only 5 of those 7 studies (71%) had bias, although the remaining 5 studies that were not funded all contained bias (100%). We additionally investigated whether following PRISMA guidelines affected the rate of bias; 9 or 12 (75%) studies reported following PRISMA guidelines, and 7 or 9 (78%) contained bias. Of the 3 studies not following PRISMA guidelines, all contained bias.

Using the AMSTAR 2 system (Table 3), we found that 8 of 12 (67%) were rated as critically low confidence, 25% (3/12) were rated as low confidence, and 8% (1/12) rated as moderate confidence. A study was classified as critically low if it contained more than 1 critical flaw and classified as low if it contained only 1 critical flaw. Of the most common critical flaws, 67% (8/12) failed to meet criteria 2 (“Did the report of the review contain an explicit statement that the review methods were established before the conduct of the review, and did the report justify any significant deviations from the protocol?”), and a further 83% (10/12) did not meet criteria 15 (“if they performed quantitative synthesis, did the review authors carry out an adequate investigation of the publication bias [small study bias] and discuss its likely impact on the results of the review?”).

**Table 3:** AMSTAR 2 Assessment of Study Quality of Reviewed Studies

AMSTAR 2 Item		Response, n	
		Yes	No
1.	Did the research questions and inclusion criteria for the review include the elements of PICO?	12	0
2.	Did the report of the review contain an explicit statement that the review methods were established before the conduct of the review, and did the report justify any significant deviations from the protocol?	4	8
3.	Did the review authors explain their selection of the study designs for inclusion in the review?	2	10
4.	Did the review authors use a comprehensive literature search strategy?	12	0
5.	Did the review authors perform study selection in duplicate?	11	1
6.	Did the review authors perform data extraction in duplicate?	7	5
7.	Did the review authors provide a list of excluded studies and justify the exclusions?	7	5
8.	Did the review authors describe the included studies in adequate detail?	10	2
9.	Did the review authors use a satisfactory technique for assessing the risk of bias in individual studies that were included in the review?	6	6
10.	Did the review authors report on the sources of funding for the studies included in the review?	0	12
11.	If meta-analysis was performed, did the review authors use appropriate methods for statistical combination of results?	8	4
12.	If meta-analysis was performed, did the review authors assess the potential impact of risk of bias in individual studies on the results of the meta-analysis or other evidence synthesis?	7	5
13.	Did the review authors account for risk of bias in primary studies when interpreting/discussing the results of the review?	10	2
14.	Did the review authors provide a satisfactory explanation for, and discussion of, any heterogeneity observed in the results of the review?	10	2
15.	If they performed quantitative synthesis, did the review authors carry out an adequate investigation of publication bias (small study bias) and discuss its likely impact on the results of the review?	2	10
16.	Did the review authors report any potential sources of conflict of interest, including any funding they received for conducting the review?	11	1

AMSTAR, A Measurement Tool to Assess Systematic Reviews, version 2; PICO, Population, Intervention, Comparison, and Outcome.

There were no substantial associations (*P* > .05) between either funding source or PRISMA adherence and the presence of spin.

## Discussion

In this study, we found at least 1 form of spin in the abstracts of 10 of 12 studies. Regarding the specific categories of spin type, misleading interpretation was identified in 58% (7/12) of the studies, misleading reporting in 67% (8/12) of the studies, and inappropriate extrapolation was identified in 50% (6/12) of the studies. On the basis of the AMSTAR 2 assessment, 92% (11/12) were categorized as either low quality or critically low quality.

The concept of spin bias has become increasingly analyzed in orthopaedic surgery. The high rate of spin (83%) found in our analysis is consistent with previous studies analyzing spin, which found it to be common in various orthopedic systematic reviews/meta-analyses. Previously Kim et al.^25^ looked at spin in systematic reviews looking at superior capsular reconstruction. These authors found spin in 100% of the 17 studies that met their inclusion criteria and found similar levels of bias. Interestingly, their study found type 5 spin, “Conclusion claims the beneficial effect of the experimental treatment despite high risk of bias in primary studies,” at a similar rate to our study (65% in their study and 58% in our study). They also found similar levels of type 9 bias, “Conclusion claims the beneficial effect of the experimental treatment despite reporting bias,” with 65% in their study and 58% in our study. One of the studies we analyzed compared hip arthroscopy outcomes for borderline hip dysplasia to PAO for hip dysplasia, and found that patients with borderline hip dysplasia did better with hip arthroscopy than those with hip dysplasia did after PAO; however, these are 2 different populations.^22^ This example further exemplifies the inherent risk of bias in some of the included studies. The indications for PAO and hip arthroscopy were likely different, and therefore making the claim that hip arthroscopy leads to better outcomes is further spinning the results. Although 2 patients may fall in the borderline hip dysplasia category based on LCEA, it is possible that there may have been other significant radiographic deformities that lead surgeons to perform PAOs versus isolated hip arthroscopy, and unsurprisingly, those patients had worse outcomes if other measures had defined the patient as having a high-grade dysplasia.

Within the spin category of misleading reporting, the most common types were type 5, “Conclusion claims the beneficial effect of the experimental treatment despite high risk of bias in primary studies,” being present 58% of the time. An example of spin type 5 bias was found in Murata et al.,^13^ where the abstract stated: “Random-effects meta-analysis indicated no statistically significant differences in postoperative patient-reported outcomes, the risk of revision surgery, or the risk of conversion to THA between patients who had FAI with or without BDDH.” However, when reviewing the included studies that made up the meta-analysis, there was a significant heterogeneity between groups, only short-term results, and no significant analysis of the bias of the included studies.

We found that misleading extrapolation was the least common category of spin bias within our study, present in 50% of the included systematic reviews and meta-analysis. The most common type present was type 8, “Conclusion extrapolates the review’s findings from a surrogate marker or a specific outcome to the global improvement of the disease,” and was found in 42% of the studies, in line with a previously reported rate of 24% by Kim et al.^25^ An example of type 8 spin occurred in Lodhia et al.^22^ when the abstract used the surrogate marker of conversion rate to total hip arthroplasty being lower in patients who underwent hip arthroscopy compared with patients who had undergone PAO alone or PAO plus hip arthroscopy to show that hip arthroscopy resulted in a global improvement in the disease.

Appropriately evaluating spin is important to the field of orthopedics given the reliance on technology and the corresponding influence of device companies in the field. When analyzing funding sources and their relationship to spin, our study did not find any significant relationship between funding source and the rate of spin present. This is consistent with previous studies that also reported no relationship between study funding and spin. In these studies, Checketts et al.^9^ found industry funding in 7 or 46 (15.2%) of their included studies on lower extremity joint trials, and Carr et al^10^ found only 1 or 43 (2.3%) of included studies on Achilles tendon repairs were industry funded. The relatively low rate of industry funding within these types of studies was also noteworthy.

Given the increasing use of^26^ and expanding indications for^27^ hip arthroscopy to manage hip pathology surgically, clinicians should maintain an awareness of spin bias in systematic reviews and meta-analyses that can influence clinical decision-making. Journals requiring direct evaluation of spin within manuscripts can help to ensure clinicians are making decisions with the best possible evidence available.

### Limitations

There are substantial limitations to the interpretation of our data because only 12 systematic reviews and meta-analyses met inclusion criteria, limiting the analysis in publication data and impact factor of the journal publishing these studies. Additionally, the small sample size may have affected the statistical significance in data analysis. Therefore, this study should be analyzed qualitatively in reporting the rates as well as types of spin found. Finally, the nature of categorizing spin bias may itself be subject to interpretation and bias. Because the spin analysis in this study was performed by multiple reviewers, we aimed to minimize this effect, but further research and validation for the methods for spin bias assessment may be needed.

## Conclusions

Spin bias is frequently encountered in the abstracts of systematic reviews and meta-analyses that evaluate outcomes after hip arthroscopy for the treatment of hip pathology in the setting of borderline hip dysplasia.

## Disclosures

The authors declare the following financial interests/personal relationships, which may be considered as potential competing interests: A.Z. reports consulting or advisory relationships with Stryker Orthopaedics, DePuy Synthes Mitek Sports Medicine, and CONMED, and serves on the Editorial Board of the *Journal of Arthroscopy.* All other authors (J.J.T., M.J.H., S.G.M., S.E.W.) declare that they have no known competing financial interests or personal relationships that could have appeared to influence the work reported in this paper.
